# Single cell biotechnology to shed a light on biological ‘dark matter’ in nature

**DOI:** 10.1111/1751-7915.12249

**Published:** 2015-01-28

**Authors:** Wei E Huang, Yizhi Song, Jian Xu

**Affiliations:** 1 Department of Engineering Science, University of OxfordParks Road, Oxford, OX1 3PJ, UK; 2 Kroto Research Institute, University of SheffieldBroad Lane, Sheffield, S3 7HQ, UK; 3 Single-Cell Center, CAS Key Laboratory of Biofuels and Shandong Key Laboratory of Energy Genetics, Qingdao Institute of Bioenergy and Bioprocess Technology, Chinese Academy of SciencesQingdao, 266101, China

It is estimated that over 99% of microorganisms have not as yet been cultivated (Whitman *et al*., 1998). These uncultured microbes are the ‘dark matter’ of the microbiological world and play important roles in natural ecosystems and the human microbiome (Lagier *et al*., 2012; Rinke *et al*., 2013); however, their ecological role and function remain largely elusive (Lasken, 2012; Li *et al*., 2012a,b). Furthermore, these uncultured bacteria represent a significant, yet largely untapped, genetic resource for use in synthetic biology for the provision of novel bioparts or biobricks, in medicine for new drug biosynthesis, in industry for robust biocatalysts and biofuel synthesis, and in environmental bioremediation for new biodegradation genes.

Metagenomics circumvents the cultivation issue by extracting the total DNA from an environmental sample, and directly sequencing it (Handelsman, 2004), and this approach has revealed an unprecedented view of the diversity and complexity of microbial communities. However, such approaches are usually unable to define or validate the specific role of individual members of the usually complex microbiota. Single cell biotechnology, which characterizes microbial cells in their native microbiota one by one, offers a new approach to study uncultured bacteria. An ideal platform is to integrate accurate and ‘contamination-free’ single cell sorting tools with powerful next-generation DNA sequencing. This will usher in a new area of single cell omics (genomics, transcriptomics, proteomics and metabolomics).

For single cell biotechnology, there are a number of key challenges: non-invasive and *in-vivo* cell analysis, the linking of cell phenotypes to specific ecological functions (e.g. substrate metabolism), overcoming limitations in measurement parameters, systematic differentiation of the given ‘state’ or phenotype of a cell and isolation of live single cells from complex samples *in-situ*.

Among the various single cell sorting techniques (Lasken, 2012; Li *et al*., 2012a,b), an emerging approach is Raman-activated cell sorting (RACS), which overcomes the requirement for external labelling. Single cell Raman spectra (SCRS) provide a label-free, non-invasive and intrinsic phenotypic profile of individual cells which can be used to characterize cell type, physiological state and cell functionalities (Huang *et al*., 2004; 2007a,b,c; Harz *et al*., 2009; Li *et al*., 2012a,b; Wang *et al*., 2014). A typical SCRS provides an intrinsic chemical ‘fingerprint’ of a single cell, and usually contains multi-parameter (> 1000 readings) including rich information on nucleic acids, protein, carbohydrates and lipids (Li *et al*., 2012a,b). Since SCRS measures the vibration of molecular bonds, it is sensitive to stable isotope compounds and SCRS undergoes Raman shift when cells incorporate stable isotope compounds (e.g. ^13^C-, ^15^N-substrates or ^2^H from heavy water D_2_O) into the cell's building blocks (e.g. DNA, lipids, protein or carbohydrate) (Huang *et al*., 2004; 2007a,b,c; Wang *et al*., 2013). SCRS offers a unique way to link cells to specific functions (e.g. C/N metabolism and metabolic activity) and to define cells of interest at a single cell level. A RACS system consists of a SCRS detection system and a cell isolation system that can be optical tweezers (Huang *et al*., 2009), a microfluidic device (Li *et al*., 2012a,b) or a single cell ejection system (Wang *et al*., 2013).

RACS would identify cells of interest and isolate them for downstream single cell omics analysis. The isolated single cells would be processed on microfluidic chips for DNA/RNA extraction and amplification. The DNA/RNA can then be quantified or sequenced to decode the genomes or transcriptomes of the particular cells. Such a platform directly establishes the links between genotype and phenotype of individual cells, thus offering unprecedented opportunities to study how variability of environmental and genetic impacts on the phenotype of single cells.

Single cell biotechnology will not only be a powerful tool to microbiology, but also herald single cell biology as a new frontier of cell biology. A single cell is the basic functional unit of life and all living organisms start from single cells. Learning how cells work by studying the individual cell is an important component of cell biology and single cell technology promotes a deeper understanding of cell biology. Recent research from the studies of single cells reveals that individual cells within the same population may differ dramatically in function, and these differences have profound biological implications, ranging from bacterial physiology to embryotic cell development, tissue differentiation, cancer cell formation and evolution.

In summary, during the next decade, just like DNA sequencing, single cell biotechnologies are expected to rapidly move into bench tops of biology laboratory, and to permeate all branches of life sciences and biotechnology. They promise to uncover fundamental biological principles and ultimately improve the diagnosis and treatment of diseases, unravel the ecological role of bacteria in soils, plants and humans, and promote the discovery of new gene functions for use in industry.

## Conflict of interest

None declared.
